# Groundwater quality and vertical electrical sounding data of the Valliyar River Basin, South West Coast of Tamil Nadu, India

**DOI:** 10.1016/j.dib.2019.103919

**Published:** 2019-04-15

**Authors:** S. Rajkumar, Y. Srinivas, Nithya C. Nair, S. Arunbose

**Affiliations:** Centre for Geotechnology, Manonmanaiam Sundaranar University, Abishekapatti, Tirunelveli, Tamilnadu, 627012, India

**Keywords:** Groundwater, Valliyar river, Water quality index, VES

## Abstract

The present study was carried out to assess the drinking water quality and aquifer characteristics of the Valliyar river basin in Kanyakumari district. 71 groundwater samples were collected and analyzed to understand the quality of water based on the index parameters. 23 vertical electrical soundings (VES) were conducted to understand the subsurface characteristics and their impact on the quality of water. The VES data suggested that the subsurface lithology consist of three distinct layers. The water quality index (WQI) showed that 25% of the samples falling under excellent water and 51% of the samples were classiﬁed as good water and another 24% belong in the poor water category.

**Table:** Specifications table

Subject area	Earth and planetary sciences
More specific subject area	Groundwater Chemistry and Hydrogeophysics
Type of data	Tables and Figures
How data was acquired	Sample collections, Field analysis, Laboratory analysis
Data format	Raw, analyzed.
Experimental factors	Groundwater samples from 71 different locations and 23 vertical electrical soundings were conducted in Valliyar river basin, Tamil Nadu, India
Experimental features	Physical and chemical parameter such as pH, TDS, EC, TH, Ca^2+^, Mg^2+^, HCO_3_^−^, Na^+^, K^+^, Cl^−^ and SO_4_^2−^ were analyzed according to APHA method. VES method using to identify aquifer resistivity, depth and thickness.
Data source location	Valliyar River Basin, Tamilnadu, India
Data accessibility	Data are available in the article
Related research article	Y. Srinivas, D. Hudson Oliver, A. Stanley Raj, N. Chandrasekar, Geophysical and geochemical approach to identify the groundwater quality in Agastheeswaram Taluk of Kanyakumari District, Tamil Nadu, India, Arab J Geosci., 8, 2015, 10647–10663 [1].

**Table:** 

**Value of the data** • The geochemical data set was used to identify the drinking water quality • The irrigation water quality (Na%, SAR, and PI) was suggesting the suitability of water. • The vertical electrical sounding data is helpful to understand the subsurface lithology, aquifer resistivity and thickness.

## 1 Data

**Fig. 1:**
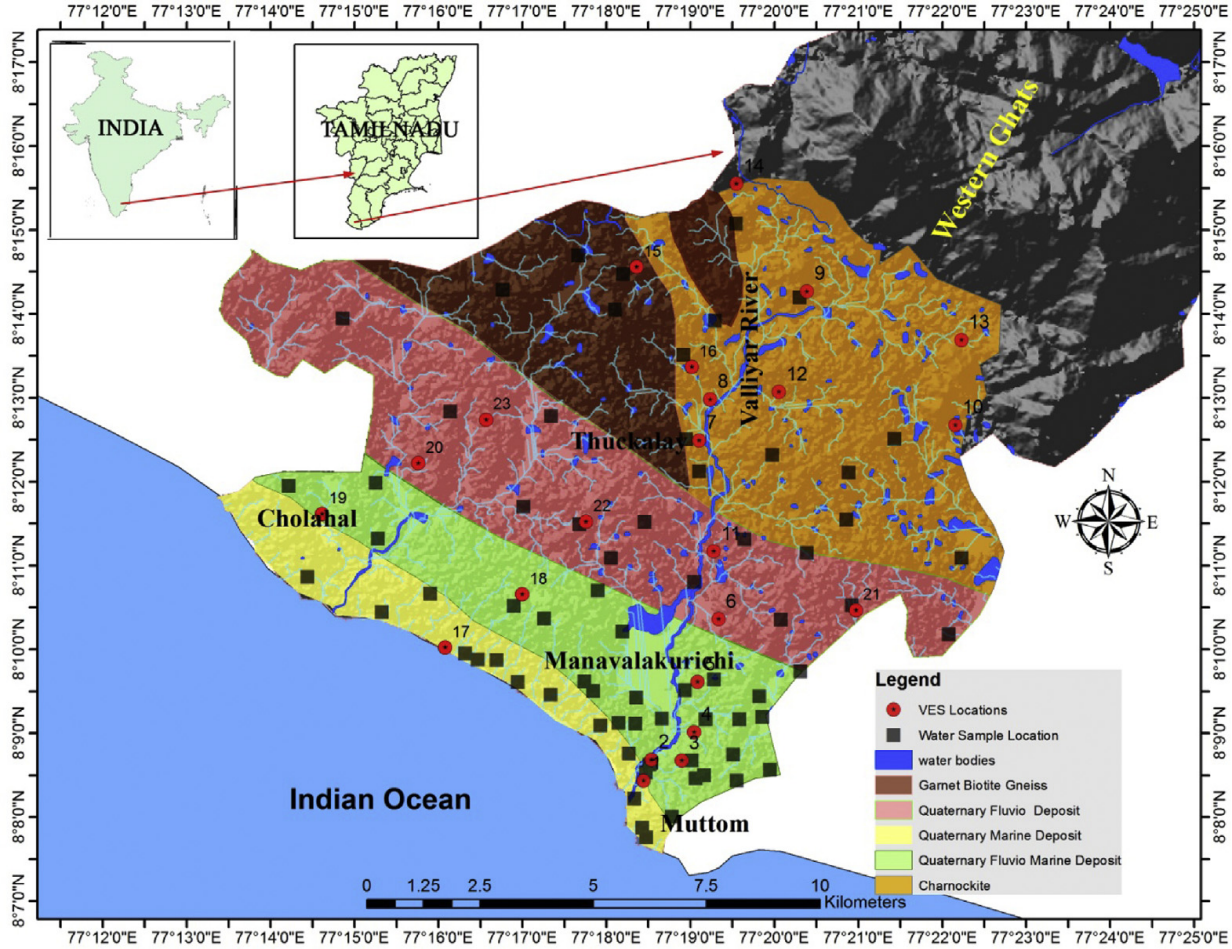
Location map of the study area.

**Fig. 2:**
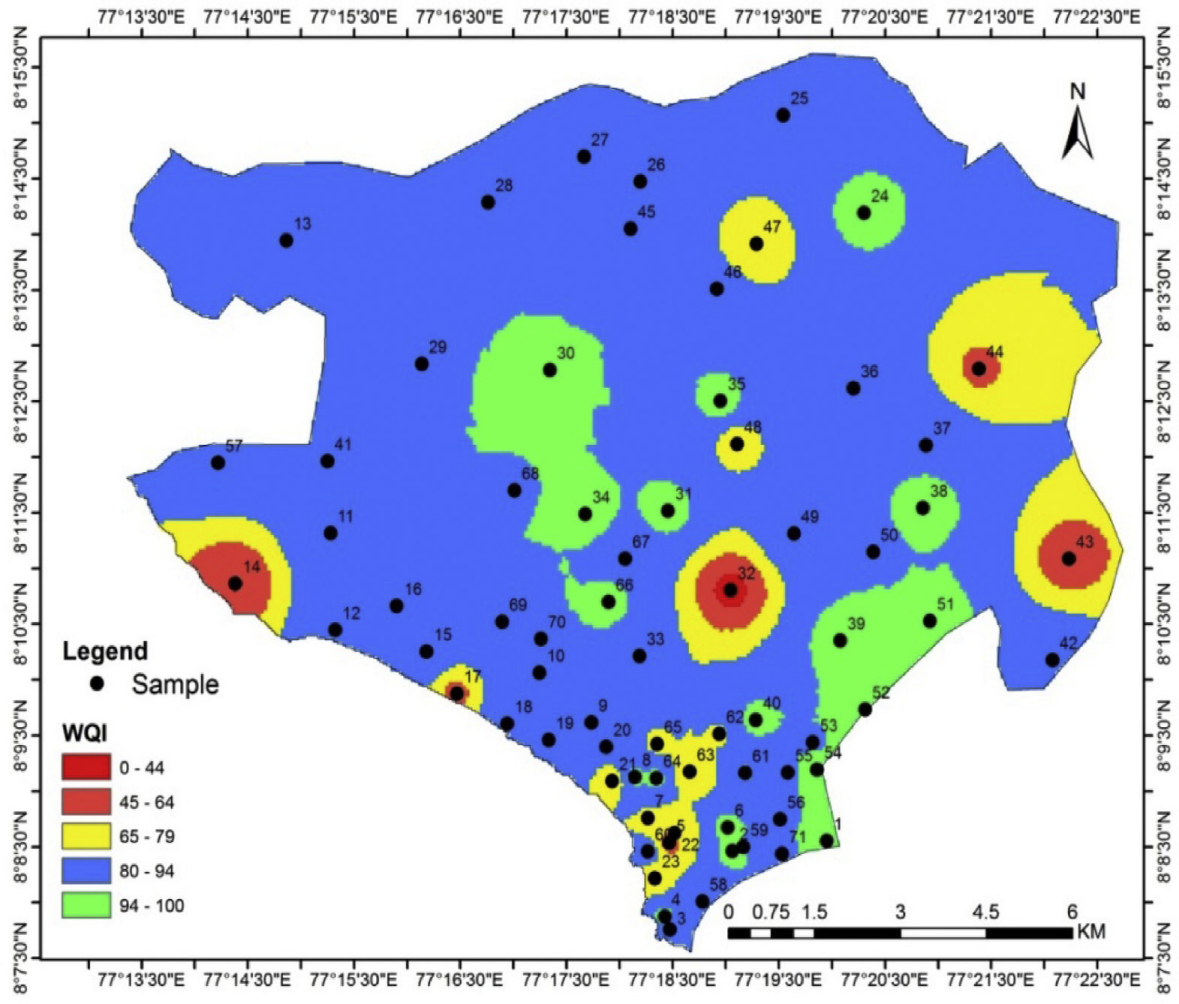
Spatial distribution of water quality index.

**Fig. 3:**
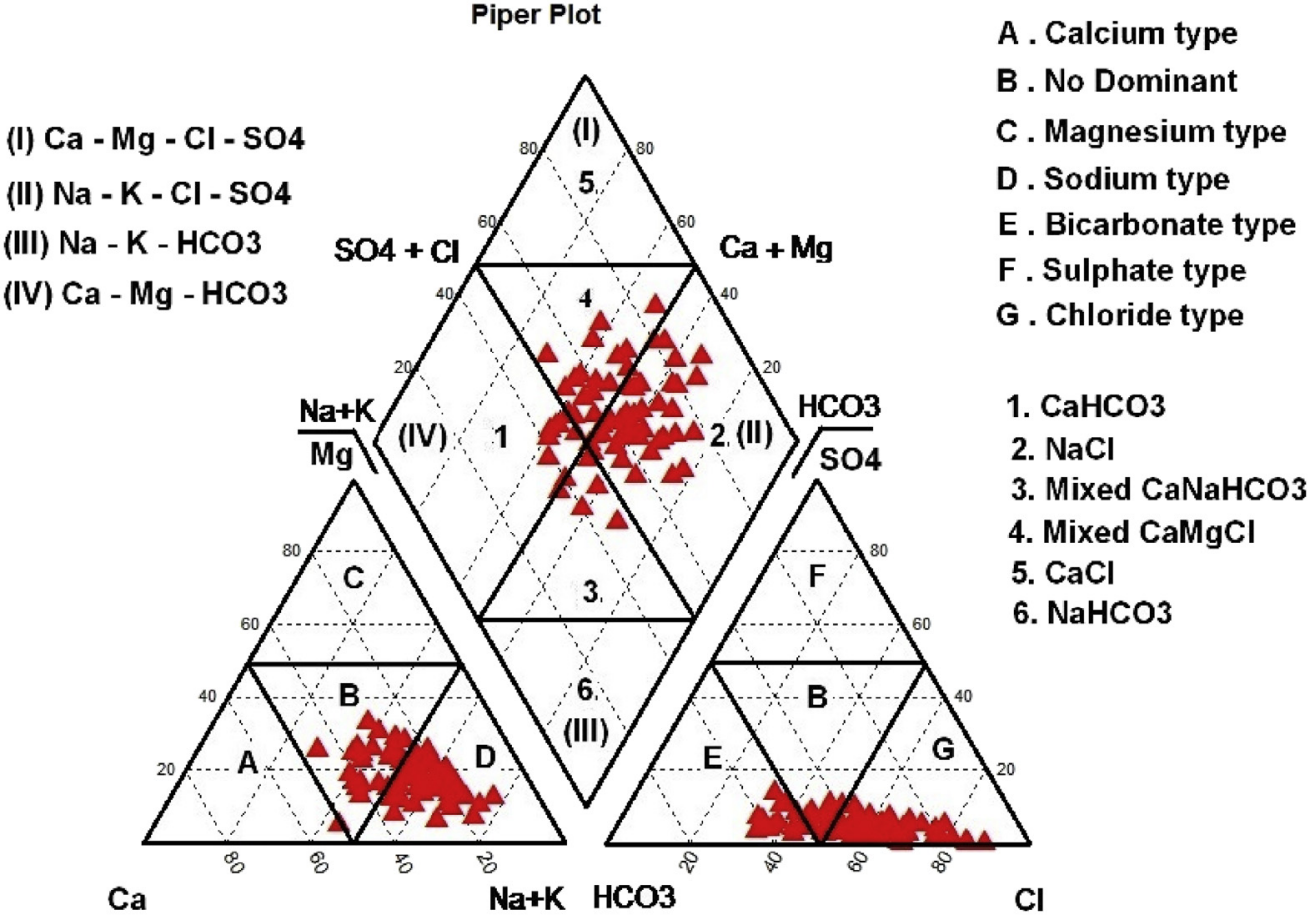
Piper diagram of groundwater in the study area.

This dataset contains six figures and five tables that represent the suitability of the groundwater for drinking and irrigation purposes of Valliyar River basin, Kanyakumari district, India. Fig. 1 shows the geochemical and geophysical data location map of Valliyar River basin. Fig. 2 shows spatial distribution of water quality index. Hydrogeochemical facies for groundwater shows in Fig. 3. Table 1 indicates data of various physio-chemical parameters such as pH, electrical conductivity (EC), total dissolved solids (TDS), Ca^2+^, Mg^2+^, K^+^, Na^+^, Cl^−^, HCO^3−^ and SO_4_^2−^ and their comparison with existing firm standards. This comparison disclose the quality of water and its suitability for drinking and irrigation needs. pH is the molar concentration of hydrogen (H) ions which express the alkaline or acidic condition of water. pH value ranges from 6.5 to 8.5 is suitable for drinking water based on WHO. EC value used to get an idea about salt enrichment in groundwater [1]. Total dissolved solids (TDS) can be taken as a measurement of dissolved inorganic salts and some organic matter in water. In this study area TDS value ranges from 36 to 1718 mg/l. Table 2 shows the water quality indices of Canadian water quality index (CWQI), Sodium percentage, Sodium Absorption Ratio and Permeability index. Irrigation parameters such as Total hardness (TH), Na%, SAR, PI, and drinking water quality index (WQI) with its classification for individual sample are given in Table 3. Presence of calcium (Ca^2+^) and magnesium (Mg^2+^) ion content determines the hardness of water. The quality criteria for determining the viability of groundwater for agricultural purposes include salinity indices, comprising Na%, SAR and PI [2]. The water quality index (WQI), was calculated to enumerate the impact of natural and anthropogenic activities. According to CWQI, the water can be classified into five types namely poor (0–44), marginal (45–64), fair (65–79), good (82–94) and excellent (94–100). Table 4 shows interpreted subsurface layer parameters (ρ, h), aquifer resistivity and aquifer thickness. It provides vertical variation of lithology as well as depth-to-aquifer and aquifer condition [3]. The aquifer thickness of the study area ranges from 2.7 to 46 m, and the average is 12 m.

**Table 1:** Physico-chemical parameters.

Sample.no	pH	EC	TDS	Ca^2+^	Mg^2+^	Na^+^	K^+^	HCO_3_^−^	Cl^−^	SO_4_^−^
1	6.84	240	154	21	12	41	5	97	68	8
2	7.15	192	123	17	8	46	2	85	57	12
3	6.82	1260	806	44	33	180	3	288	317	11
4	6.98	487	312	20	11	67	4	139	99	8
5	6.45	2093	1340	104	51	320	4	216	589	58
6	7.5	132	84	13	2	36	2	57	39	8
7	6.87	937	600	84	40	101	5	309	191	18
8	7.3	540	346	42	13	76	3	166	107	9
9	6.22	484	310	24	11	81	1	69	149	7
10	5.65	387	248	12	6	68	6	55	103	11
11	5.78	160	102	10	4	26	2	43	39	7
12	5.59	501	321	21	10	87	1	103	117	14
13	5.55	56	36	12	2	14	2	37	25	8
14	5.41	2249	1439	88	55	380	18	149	781	7
15	6.09	226	145	22	8	44	3	67	78	9
16	5.86	138	88	8	2	38	3	49	50	9
17	5.82	1600	1024	54	28	190	86	137	486	5
18	6.36	605	387	44	18	46	8	117	128	8
19	6.02	393	252	24	10	71	6	109	104	11
20	5.78	656	420	36	12	118	16	167	170	9
21	6.31	908	581	36	23	128	39	122	284	7
22	5.86	1145	733	60	32	116	21	176	224	36
23	6.61	2070	1325	136	96	194	6	390	540	6
24	6.68	278	178	16	11	46	6	97	89	6
25	6.71	775	496	62	24	50	51	246	114	11
26	6.3	486	311	12	10	81	44	98	128	8
27	6.59	258	165	20	6	44	15	85	78	8
28	6.35	508	325	36	13	71	31	153	135	8
29	6.32	203	130	18	7	39	0.5	91	50	7
30	6.8	393	252	28	19	73	5	165	135	8
31	6.81	118	76	18	6	12	2	51	28	8
32	6.6	2685	1718	140	102	410	31	299	841	28
33	7.6	573	367	40	21	64	43	226	126	17
34	7.51	159	102	20	5	22	4	61	39	9
35	6.87	197	126	24	7	25	3	91	36	8
36	6.28	112	72	16	4	18	4	71	22	7
37	6.32	432	276	40	11	67	3	214	67	10
38	6.8	99	63	12	3	14	2	41	21	5
39	6.75	290	186	22	8	57	0	139	50	8
41	6.3	286	183	24	12	36	0	119	55	8
42	5.65	379	243	20	17	61	9	85	121	7
43	6.62	1860	1190	108	78	189	65	354	472	11
44	6.35	1230	787	64	48	143	14	122	433	7
45	6.22	262	168	20	11	34	7	99	46	14
46	6.19	553	354	24	17	67	29	108	124	16
47	6.18	855	547	63	32	102	25	155	227	21
48	6.63	1067	683	60	28	114	51	198	242	28
49	7.04	470	301	24	9	60	11	103	85	13
50	6.35	211	135	13	7	27	2	86	28	8
51	6.6	168	108	20	9	24	2	74	50	10
52	6.63	71	45	11	5	13	2	37	25	8
53	5.84	277	177	36	2	34	4	75	64	8
54	6.7	357	228	38	10	39	10	128	67	12
55	5.75	129	83	13	2	22	2	37	32	9
56	5.39	148	95	14	4	20	3	38	36	7
57	5.92	308	197	12	6	50	5	61	75	9
58	5.94	365	234	16	7	58	5	54	89	12
59	5.62	591	378	20	10	94	7	98	149	9
60	6.28	179	115	13	4	24	3	57	36	8
61	5.89	872	558	48	28	114	6	103	236	19
62	6.26	859	550	48	29	106	21	173	199	17
63	6.34	1698	1087	56	37	193	49	254	389	24
64	6.65	363	232	26	11	52	2	109	77	10
65	6.25	929	595	50	19	111	31	173	187	12
66	6.5	98	63	11	2	17	4	49	21	9
67	6.16	466	298	28	14	67	13	118	110	10
68	5.5	98	63	8	2	14	3	28	21	6
69	5.38	119	76	10	2	21	3	52	19	11
70	5.21	142	91	8	2	26	6	57	23	9
71	5.71	404	259	20	9	38	7	83	62	10

(unit for all parameters are mg/l except pH (on scale) and EC (μs/cm)).

**Table 2:** Summary of water quality indices for drinking and irrigation [4], [5], [6].

Indices	Acronym	Formula
Canadian Water Quality index	CWQI	$CWQI\;=\;\;100-\sqrt{(}F_{1}^{2}+F_{2}^{2}+F_{3}^{2})/1.732$
Sodium percentage	Na%	$Na\%=Na^{+}\text{x}\;\;100/\;\left[Ca^{2+}\;+Mg^{2+}\;+Na^{+}+K^{+}\right]$
Sodium Absorption Ratio	SAR	$\text{SAR}\;=\;\text{Na}+/\left[\left({\text{Ca}}^{2+}+{\text{Mg}}^{2+}\right)/2\right]0.5$
Permeability index	PI	$PI=\left[Na^{+}+HCO_{3}^{\left(0.5\right)}\right]\;\text{x}\;100/\;\left[Na^{+}\;+Ca^{2+}\;+Mg^{2+}\right]$

**Table 3:** Irrigation parameters and classification of drinking water quality index (WQI) [7], [8].

S. no	TH	SAR	PI	Na%	WQI	Quality Category
1	102	1.8	80	48	100	Excellent
2	76	2.3	91	58	100	Excellent
3	245	5.0	79	62	84	Good
4	97	3.0	91	61	100	Excellent
5	470	6.4	68	60	52	Marginal
6	41	2.5	106	67	100	Excellent
7	374	2.3	56	38	78	Fair
8	160	2.6	76	51	100	Excellent
9	106	3.4	81	63	93	Good
10	54	4.0	97	74	93	Good
11	43	1.7	99	58	93	Good
12	93	3.9	90	67	93	Good
13	40	1.0	98	45	93	Good
14	445	7.8	71	66	43	Poor
15	89	2.0	80	53	93	Good
16	29	3.1	114	75	93	Good
17	252	5.2	73	68	59	Marginal
18	184	1.5	60	37	93	Good
19	100	3.1	87	62	93	Good
20	138	4.4	86	67	85	Good
21	186	4.1	75	64	67	Fair
22	280	3.0	63	50	69	Fair
23	734	3.1	47	37	64	Marginal
24	84	2.2	89	56	100	Excellent
25	256	1.4	57	41	83	Good
26	70	4.2	97	77	79	Fair
27	77	2.2	90	60	92	Good
28	144	2.6	78	57	82	Good
29	75	2.0	91	53	93	Good
30	150	2.6	78	52	100	Excellent
31	71	0.6	74	29	100	Excellent
32	768	6.4	60	55	34	Poor
33	187	2.0	72	51	85	Good
34	70	1.1	83	43	100	Excellent
35	88	1.2	81	40	100	Excellent
36	58	1.0	96	43	93	Good
38	42	0.9	98	44	100	Excellent
39	90	2.6	93	58	100	Excellent
40	106	1.4	80	40	100	Excellent
41	110	1.5	79	42	93	Good
42	119	2.4	76	55	93	Good
43	591	3.4	53	46	54	Marginal
44	356	3.3	57	48	62	Marginal
45	96	1.5	81	46	93	Good
46	129	2.6	77	59	83	Good
47	288	2.6	59	47	69	Fair
48	267	3.0	66	54	72	Fair
49	98	2.6	85	60	93	Good
50	61	1.5	99	50	93	Good
51	87	1.1	77	39	100	Excellent
52	47	0.8	89	40	100	Excellent
53	100	1.5	74	44	93	Good
54	136	1.5	71	42	100	Excellent
55	41	1.5	98	55	93	Good
56	52	1.2	87	48	93	Good
57	55	2.9	97	68	93	Good
58	68	3.1	89	66	93	Good
59	90	4.3	91	70	93	Good
60	50	1.5	98	53	93	Good
61	234	3.2	65	52	78	Fair
62	238	3.0	67	52	77	Fair
63	294	4.9	73	62	64	Marginal
64	109	2.2	81	52	100	Excellent
65	205	3.4	73	58	76	Fair
66	38	1.2	109	53	100	Excellent
67	130	2.6	78	56	85	Good
68	28	1.2	110	55	93	Good
69	34	1.6	115	59	93	Good
70	29	2.1	122	69	92	Good
71	85	1.8	84	52	93	Good

Where, TH – Total Hardness; SAR – Sodium Absorption Ratio; PI – Permeability Index; WQI - Water Quality Index; Na % - Sodium Percentage.

**Table 4:** Interpreted subsurface layer parameters, aquifer resistivity and thickness from vertical electrical sounding data.

VES.no	Subsurface layer parameter							Aquifer resistivity	Aquifer thickness
	ρ1	ρ2	ρ3	ρ4	h1	h2	h3		
VES1	166	18	125	_	1.74	8.4	_	18	8.4
VES2	105	8	26.5	2241	3.18	5.2	31	8	5.2
VES3	150	40	23	1534	1	7.3	60	23	60
VES4	119	58	276	8	11.4	14	28.6	8	_
VES5	28	169	11.5	13319	2	3	9.6	11.5	9.6
VES6	56.6	7	294	_	1.2	4	_	7	4
VES7	60	10	34	3242	4.9	3.7	38	10	3.7
VES8	262	29	861	_	2.6	4.5	_	29	4.5
VES9	189	29	1095	32	1.7	12.6	20	29	12.6
VES10	153	17.6	7446	_	1.6	11	_	17.6	11
VES11	50	5.7	61	_	1.4	2.7	_	5.7	2.7
VES12	82	6.5	498	_	2.5	5.36	_	6.5	5.4
VES13	102	3.7	8264	_	2	5.2	_	3.7	5.2
VES14	52	112	44	392	1	1.7	3.5	44	3.5
VES15	44	23.8	14.8	5205	1	8.6	11.3	14.8	11.3
VES 16	181	72.2	217	1982	2.9	5.4	15.4	72.2	5.5
VES17	40.4	8.17	96	0.37	2.15	5.13	15.1	0.372	_
VES 18	456	133	553	8.44	1.42	12.2	44.4	8.44	_
VES19	233	68.8	1180	2.39	6.6	11	13.3	2.39	_
VES20	48.5	36	88.6	2329	1	7.3	15.4	36	7.3
VES21	212	81	27.2	2742	1	3.73	46.3	27.2	46.3
VES22	296	134	372	_	2.54	7.34	_	134	7.34
VES23	86.9	977	181	91.5	1	1.1	30.4	91	_

Where, ρ (Ω⋅m) and h (m) means resistivity and thickness of the subsurface layers.

## 2 Materials and methods

### 2.1 Groundwater sample collection

Groundwater samples were randomly collected from 71 open and bore wells during January 2015. For the sample collection, high density polyethylene bottles were used. The bottles are immediately sealed after the sample collection to avoid the reaction with the atmosphere. The sample bottles were labeled systematically. The collected samples were analyzed in the laboratory for various physicochemical parameters. During sample collection, handling, preservation and analysis standard procedure recommended by the American public health organization [9] were followed to ensure data quality and consistency.

### 2.2 Field analysis (physical parameters)

The physical parameters such as pH, Electrical Conductivity (EC) were measured in-situ using Hanna water quality meter (HI-9828, USA). The Total Dissolved Solids (TDS) were calculated by multiplying the electrical conductivity by a factor of 0.64 [5].

### 2.3 Laboratory analysis (chemical parameters)

The major ions(Ca^2+^, Mg^2+^, Na^+^, K^+^, HCO_3_^−^, SO_4_^2−^, Cl^−^) were analyzed in the laboratory using the standard methods suggested by the American Public Health Association (APHA, 1995). Among the analyzed ions, sodium (Na^+^) and potassium (K^+^) were determined by using flame photometer. Calcium (Ca^2+^), magnesium (Mg^2+^), bicarbonate (HCO_3_^−^) and chloride (Cl^−^) were analyzed by volumetric methods and sulphate (SO_4_^2−^) were estimated by using the spectro-photometer. The concentration of Calcium (Ca^2+^) and magnesium (Mg^2+^) ions in the groundwater were estimated by ethylene diamine tetra acetic acid (EDTA) titration. The bicarbonate (HCO_3_^−^) ions in the groundwater samples were determined by using acid titration method, in which the sulphuric acid with 0.01 N is used. Chloride (Cl^−^) ion concentration is calculated using argentometric (AgNO_3_) titration. Sodium and potassium content were sorted out using flame photometer instrument (DEEP VISION, Model- 381). The amount of sulphate ions was found using the UV–Visible photometer.

To measure WQI a set of 11 physical and chemical parameters such as pH, EC, TDS, TH, Ca^2+^, Mg^2+^, Na^+^, K^+^, HCO_3_^−^, Cl^−^, and SO_4_^2−^ were resolved. The analysis for water quality index has been done with the help of Canadian Water Quality index (CWQI) programmed excel software [10]. ArcGIS10.1 software was used for spatial analysis of various physico-chemical factors. An Inverse Distance Weighted (IDW) technique was used to interpolate the data spatially and enumerated the value for each grid node by inspecting the encompassing data points that lie within a user defined search area.

### 2.4 Hydrogeochemical facies

The geochemical histories and flow pattern of groundwater can be determined by hydrogeochemical facies interpretation. The changes in groundwater quality within an aquifer can be understood by plotting the concentrations of dominant ions in the piper tri-linear diagram [11]. This diagram mainly consist of two triangle shaped fields each represents the composition of cations and anions, and a diamond shaped field represents composition of both cations and anions present in the groundwater. The classification of hydrogeochemical facies for groundwater plotted by piper trilinear diagram is shown in (Fig. 3). Most of the water samples fall in the NaCl segment followed by mixed CaMgCl > CaNaHCO_3_> CaHCO_3_.

### 2.5 Vertical electrical sounding

To understand the subsurface lithology and to study the groundwater potentiality within Valliyar river basin, 23 vertical electrical sounding (VES) surveys were carried out. That data were processed and interpreted manually using IPI2WIN software. The interpreted VES data reveals that the study area consist of three to four geoelectrical layers with different curve types.
