# Supplementary material for: Groundwater quality and vertical electrical sounding data of the Valliyar River Basin, South West Coast of Tamil Nadu, India
Source: Data Brief. 2019 Apr 15;24:103919. doi: 10.1016/j.dib.2019.103919 (PMC6487365; doi:10.1016/j.dib.2019.103919)
Supplement: Multimedia component 1 [file mmc1.doc]

Date – 29/03/2019

From

**S.Rajkumar**

Research Scholar

Centre for Geotechnology

Manonmaniam Sundaranar University

India

To

The Editor in Chief

Data in Brief

Dear sir/Madam

Sub- Conflict of interest for research Article – reg

The authors declare no conflict of interest connected with this manuscript entitled “**Groundwater quality and vertical electrical sounding data of the Valliyar River Basin, South West Coast of Tamil Nadu, India”.**

Thanking you

Yours faithfully

(**S.Rajkumar**)
