# Supplementary figures and images for: Groundwater quality and vertical electrical sounding data of the Valliyar River Basin, South West Coast of Tamil Nadu, India
Source: Data Brief. 2019 Apr 15;24:103919. doi: 10.1016/j.dib.2019.103919 (PMC6487365; doi:10.1016/j.dib.2019.103919)

VES 1
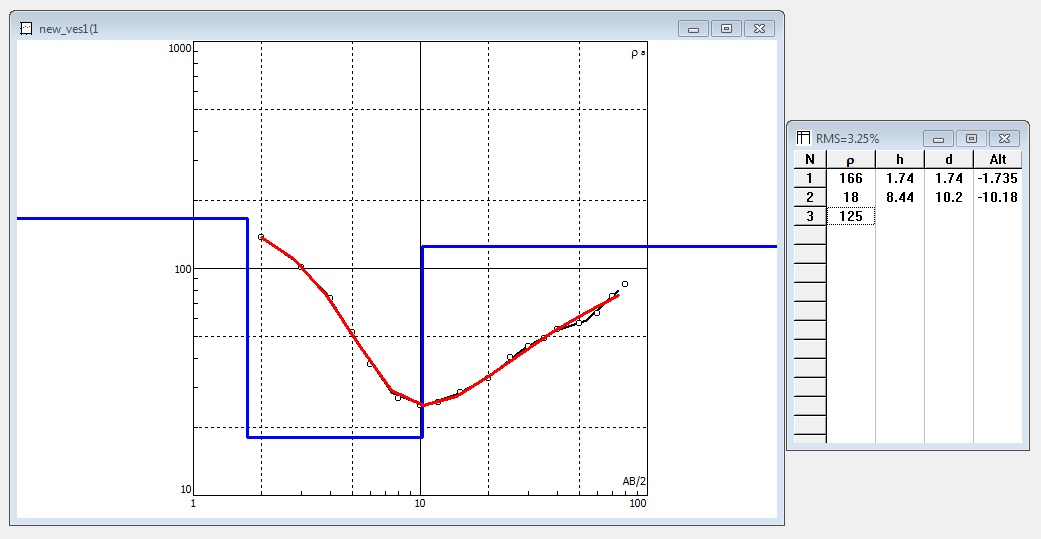


VES 2


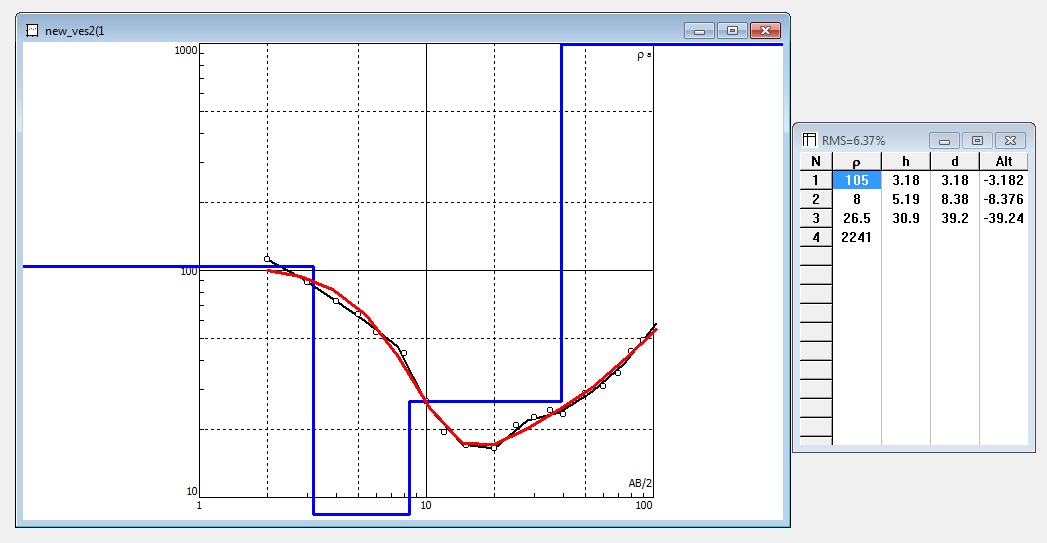


VES 3


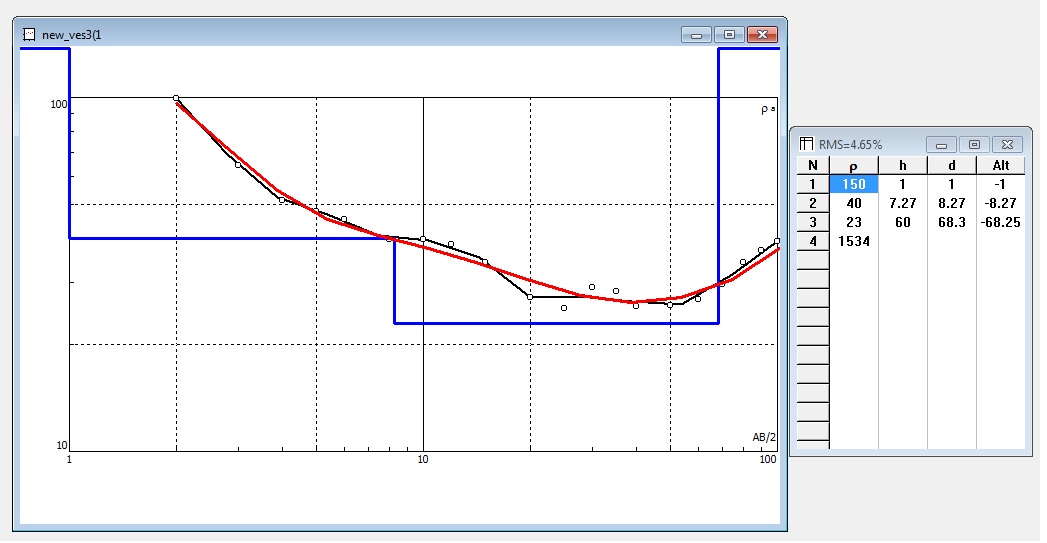


VES 4


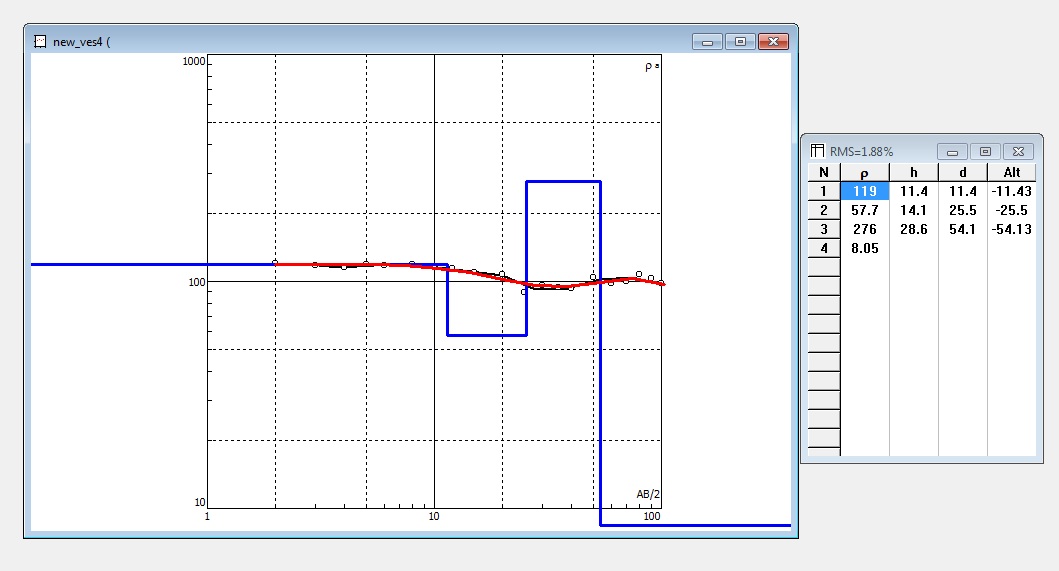


VES 5


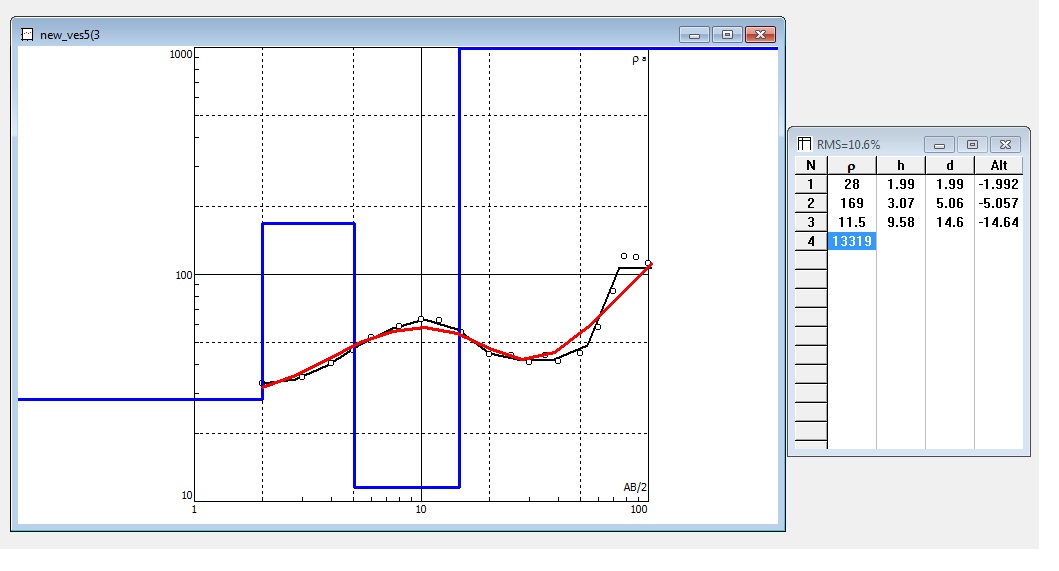


VES 6


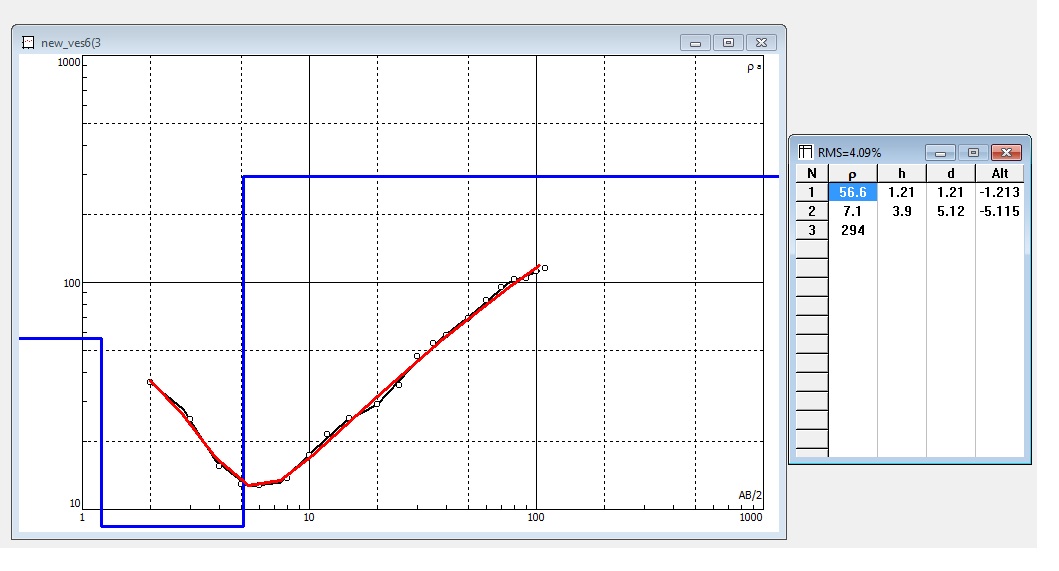


VES 7


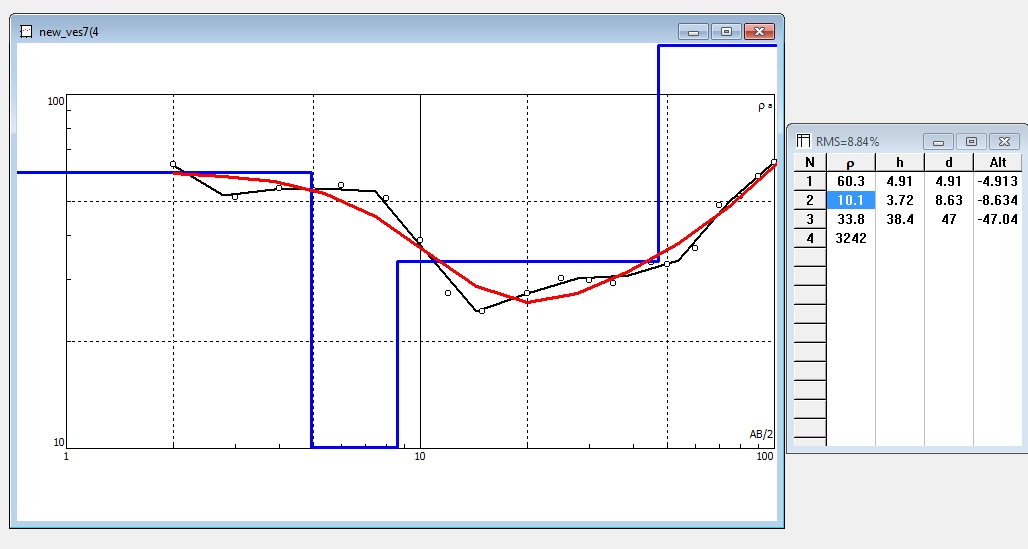


VES 8


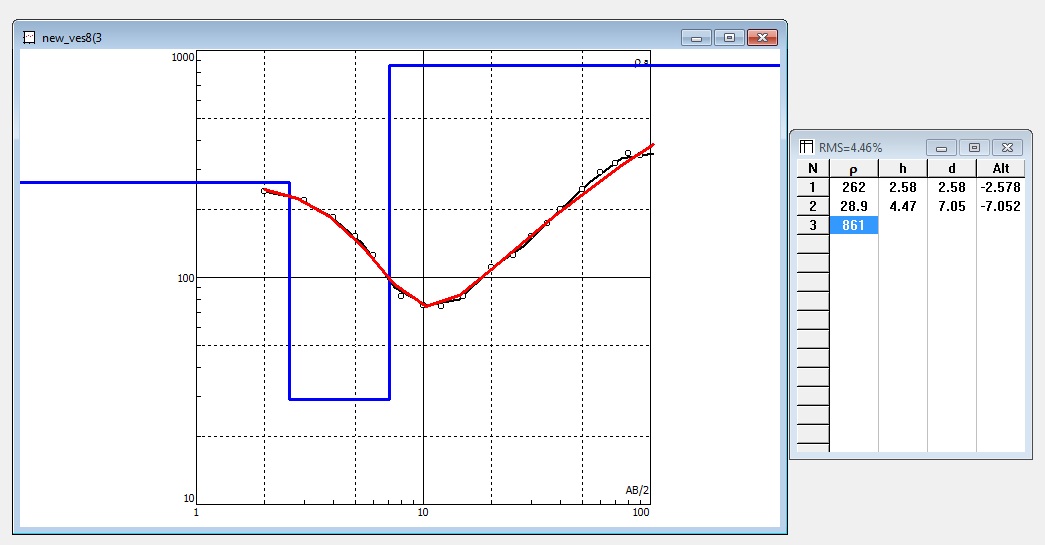


VES 9


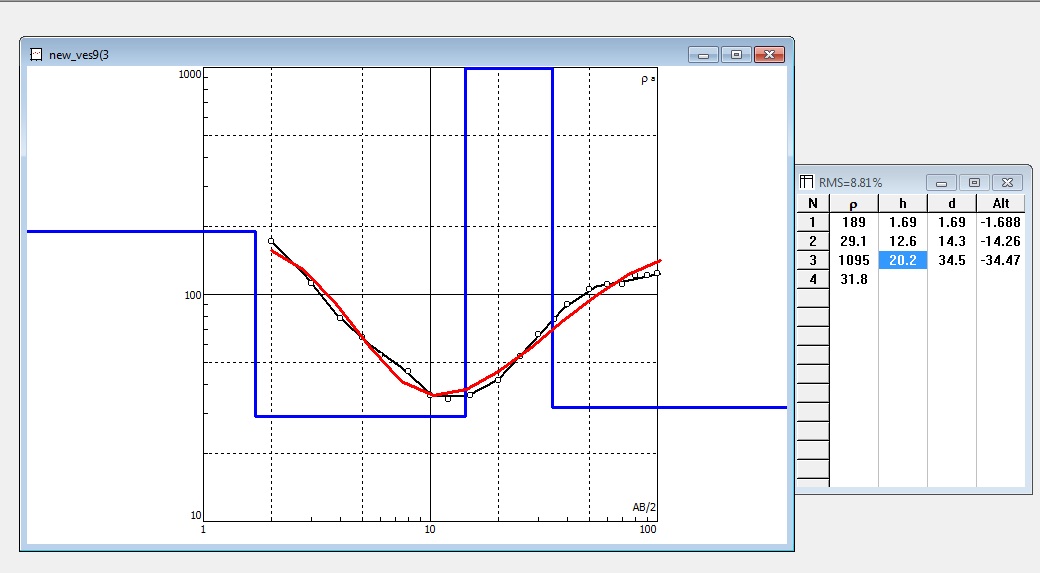


VES 10


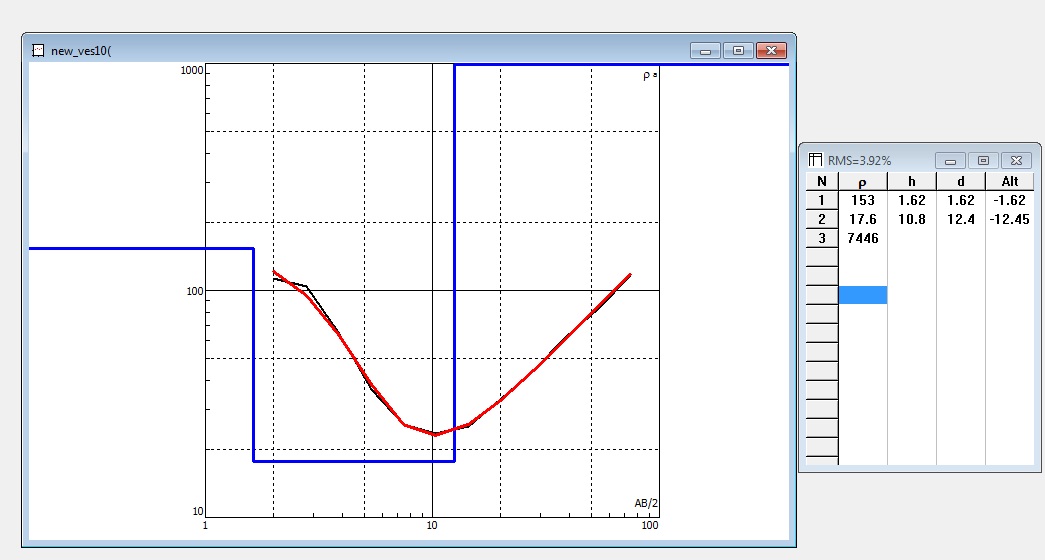


VES 11


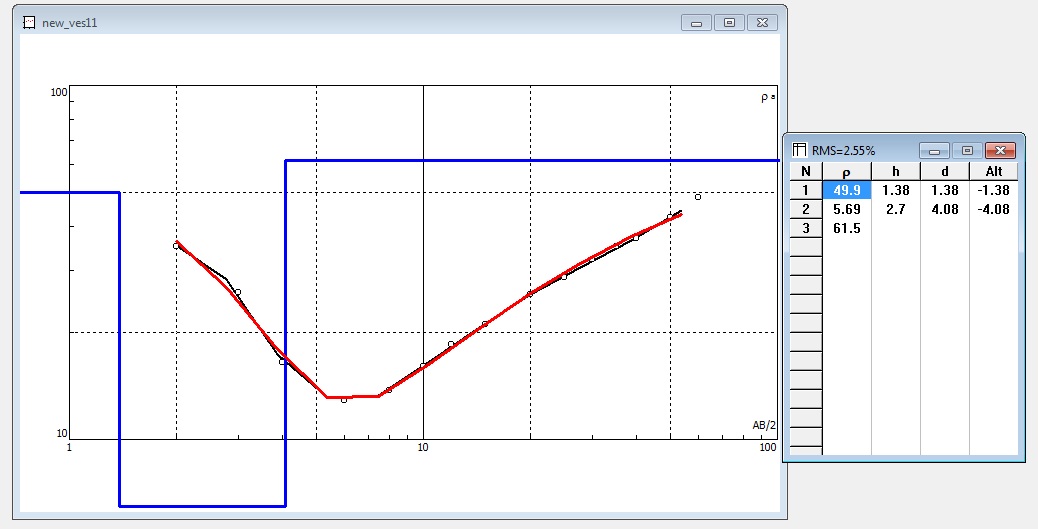


VES 12


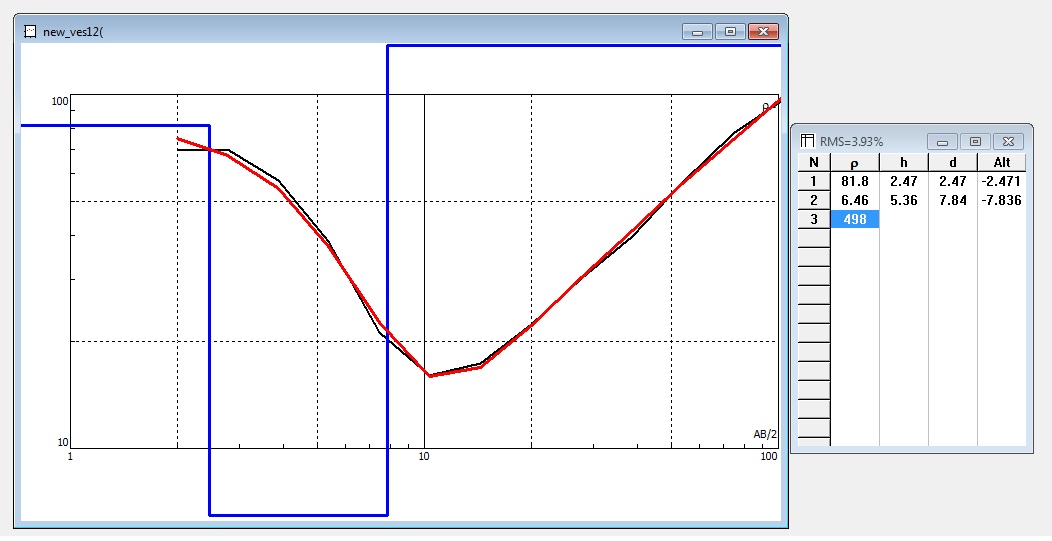


VES 13


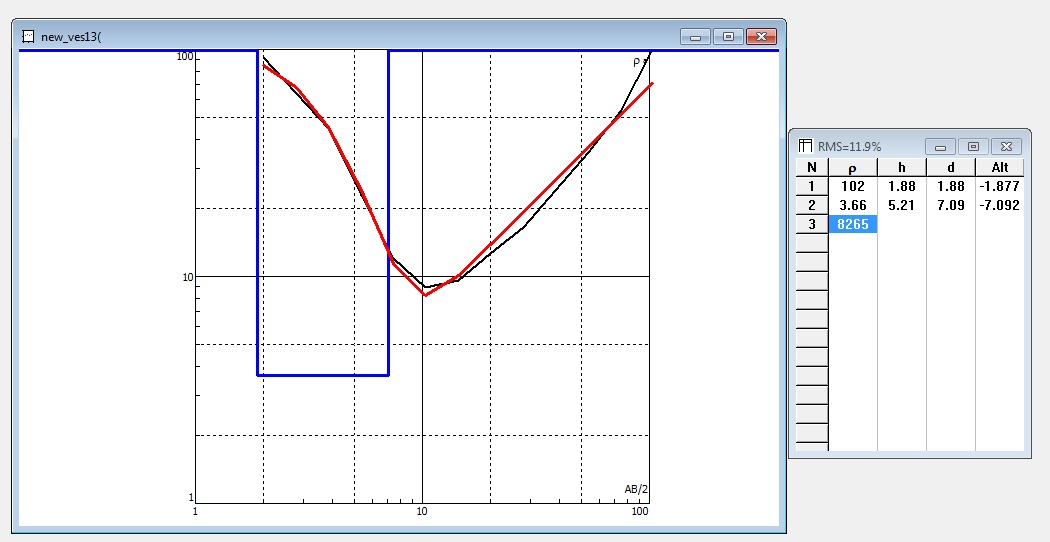


VES 14


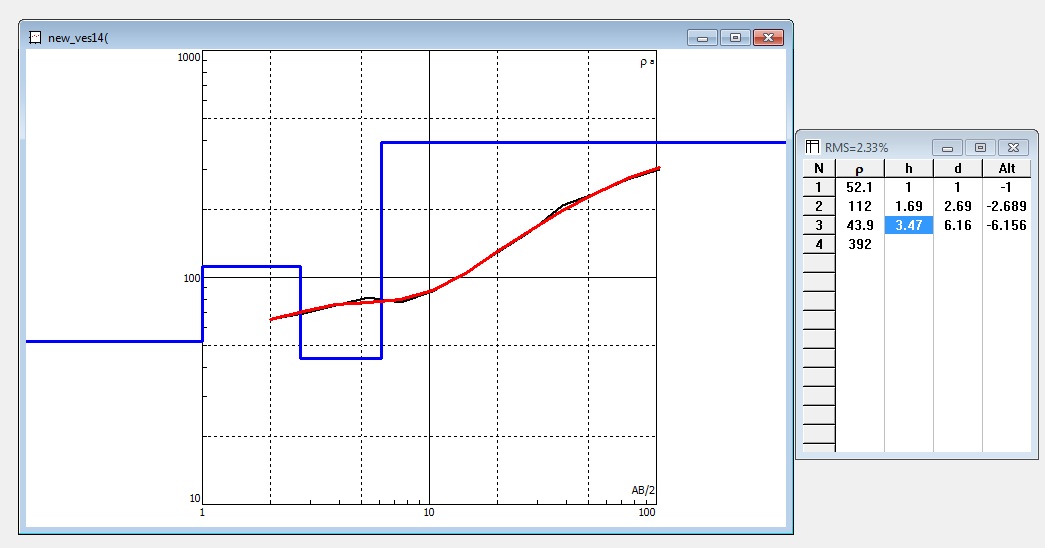


VES 15


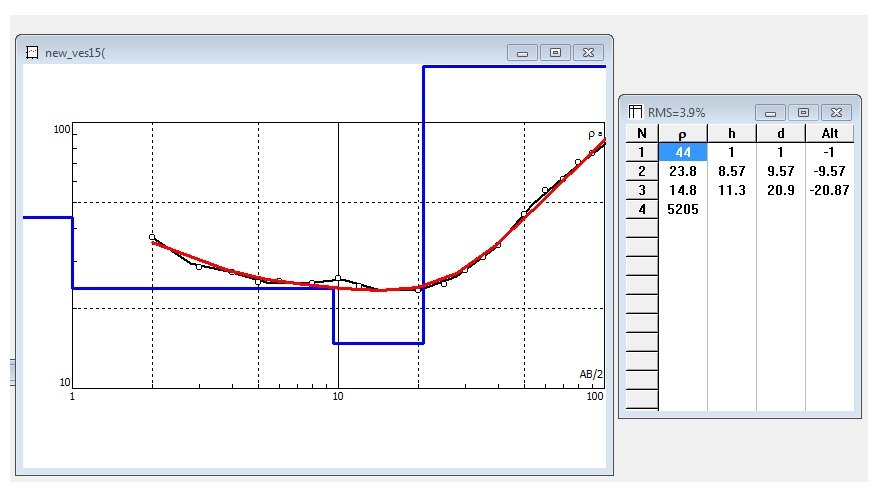


VES 16


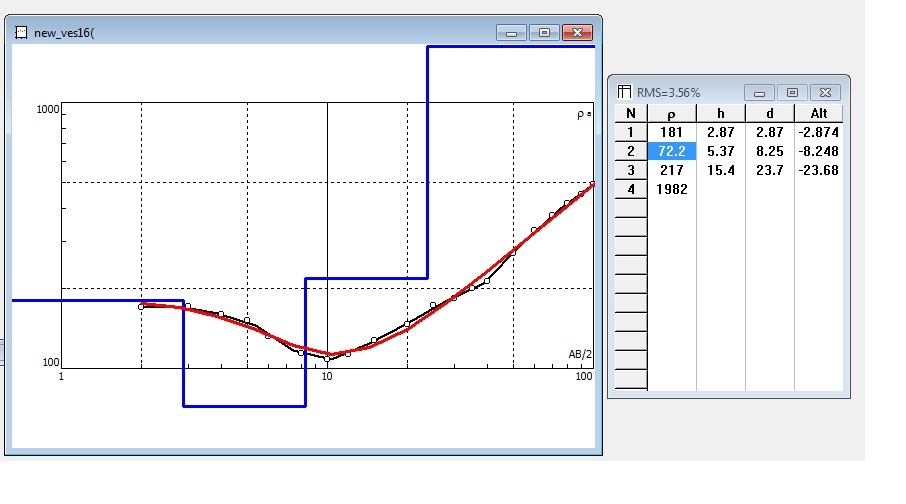


VES 17


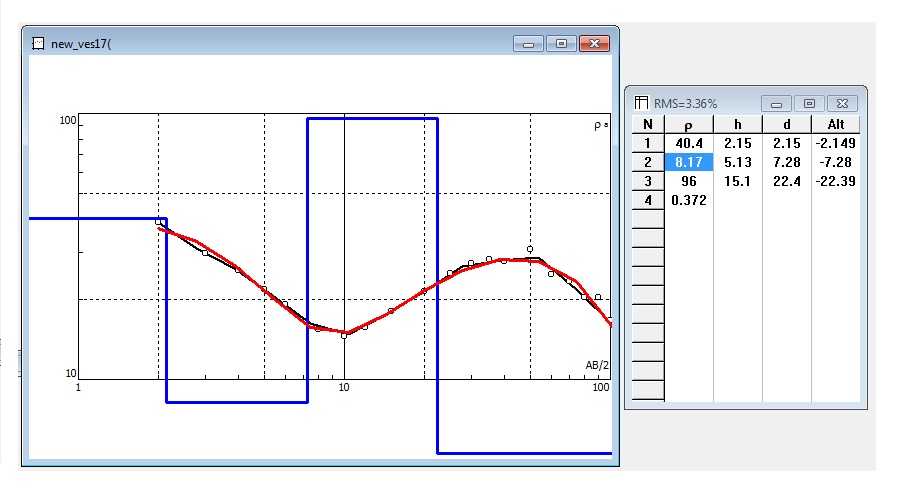


VES 18


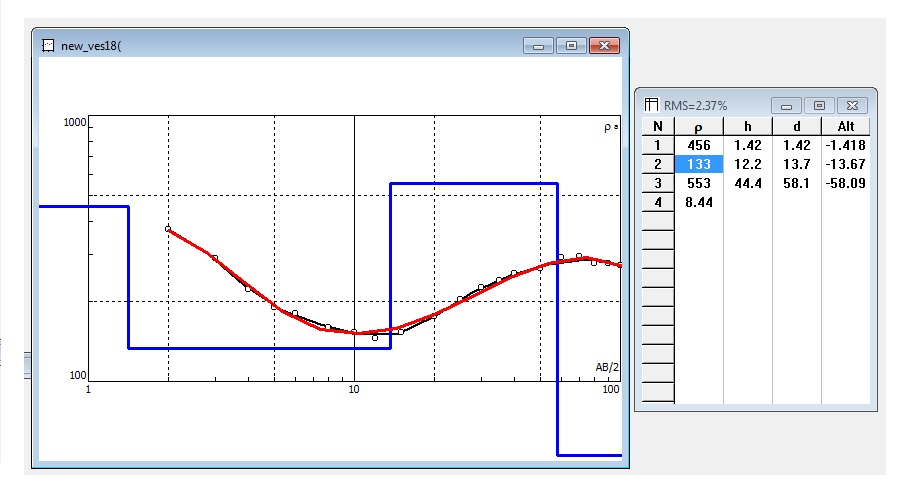


VES 19


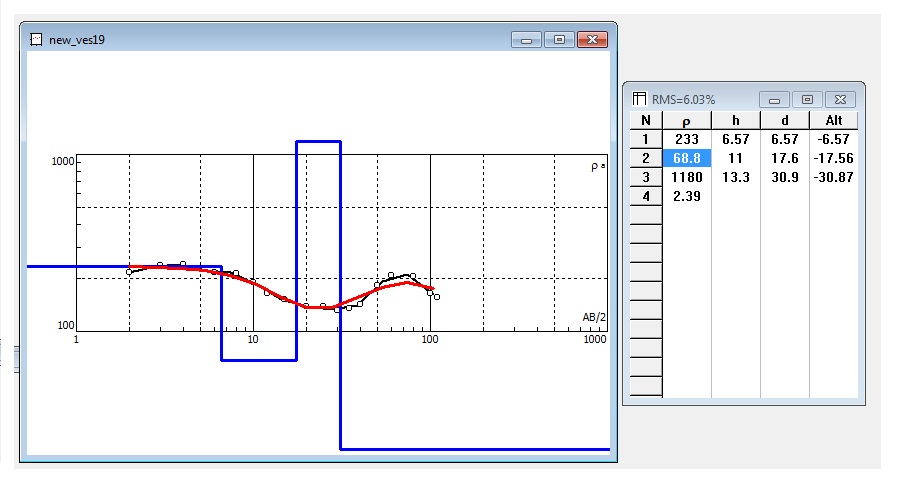


VES 20


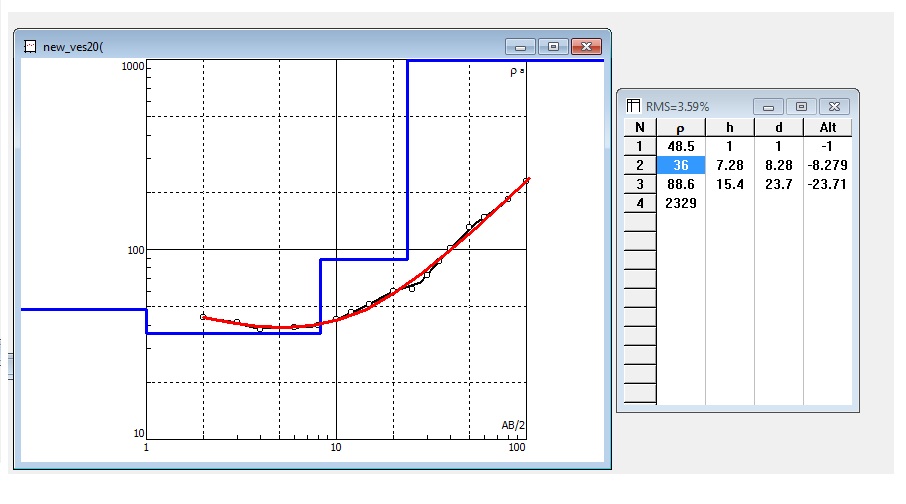


VES 21


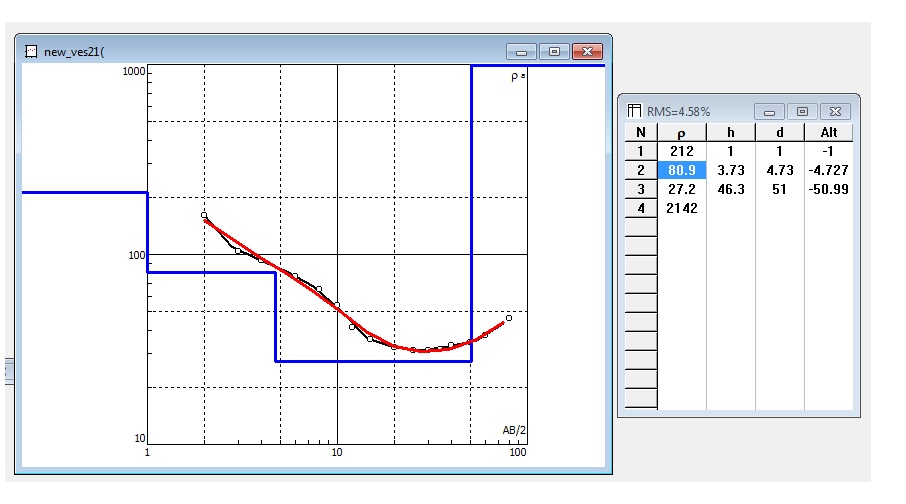


VES 22


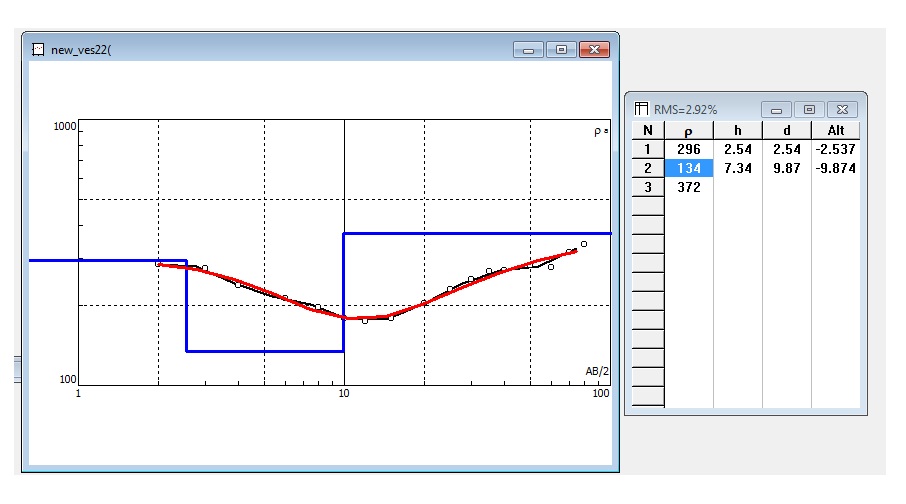


VES 23


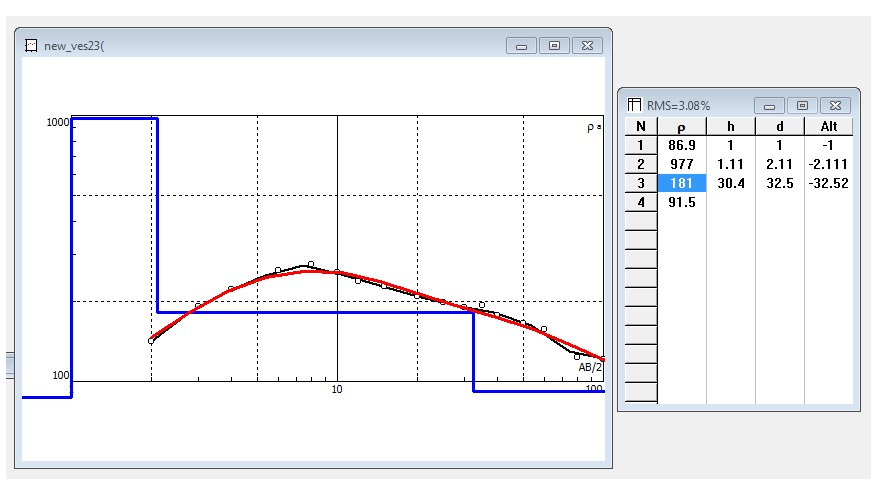

Supplement: Supplementary file 2 — Multimedia component 2 [file mmc2.docx]
